# Supplementary material for: A deeply conserved protease, acylamino acid-releasing enzyme (AARE), acts in ageing in Physcomitrella and Arabidopsis
Source: Commun Biol. 2023 Jan 17;6:61. doi: 10.1038/s42003-023-04428-7 (PMC9845386; doi:10.1038/s42003-023-04428-7)
Supplement: Supplementary file 3 — Description of Additional Supplementary Files [file 42003_2023_4428_MOESM3_ESM.pdf]

Description of Additional Supplementary Files

File name: Supplementary Data 1
Description: List of primers used to assemble KO and KI constructs and primers used to screen for transgenic plants in Physcomitrella.

File name: Supplementary Data 2
Description: List of gene accession numbers for all sequences used for phylogenetic analysis and corresponding subcellular localization predictions.

File name: Supplementary Data 3
Description: List of primers used to screen possible T-DNA mutant plants in Arabidopsis.

File name: Supplementary Data 4
Description: List of accession numbers for the International Moss Stock Center (IMSC) of all transgenic Physcomitrella lines.

File name: Supplementary Data 5
Description: List of primers used for qPCR on genomic DNA to determine copy numbers of KO constructs in transgenic Physcomitrella lines.

File name: Supplementary Data 6
Description: List of primers used to assemble the constructs for transient localization analysis in Physcomitrella protoplasts.

File name: Supplementary Data 7
Description: Spectrum report of the revised database search of the anti-Arg(Nt) IP published in Hoernstein et al., 2016. (doi: 10.1074/mcp.M115.057190).

File name: Supplementary Data 8
Description: This table contains all numeric source data used to generate the graphs in Fig. 3b, Fig. 3e-g, Fig. 5b, Fig. 6c, Fig. 6e, Fig. S1, Fig. S3c, Fig. S4j-p, Fig. S5, Fig. S6, Fig. S8d, Fig. S8e and Fig. S8g.
